# Greenhouse and field evaluation of isoxaflutole for weed control in maize in China

**DOI:** 10.1038/s41598-017-12696-7

**Published:** 2017-10-04

**Authors:** Ning Zhao, Lan Zuo, Wei Li, Wenlei Guo, Weitang Liu, Jinxin Wang

**Affiliations:** Key Laboratory of Pesticide Toxicology and Application Technique, College of Plant Protection, Shandong Agricultural University, Tai’an, 271018 China

## Abstract

Greenhouse and field studies were conducted to provide a reference for pre-emergence (PRE) application of isoxaflutole on maize in China. In greenhouse study, the isoxaflutole PRE application at 30 g active ingredient (a.i.) ha^−1^ could effectively control large numbers of weeds, especially some large-seeded broadleaves, tested in this study. The tolerance results indicated 21 maize hybrids showed different responses to isoxaflutole under greenhouse conditions. In 2015 and 2016, field experiments were conducted to determine and compare the weed control efficacy and safety to Zhengdan 958 maize with 6 herbicide treatments. In both years, isoxaflutole PRE at 100 to 250 g a.i. ha^−1^ was sufficient to provide satisfactory full-season control of the dominant common broadleaf and grass weeds in the field. Temporary injury to maize was observed with isoxaflutole treatments of 125, 150, and 250 g a.i. ha^−1^ in both years, but plants recovered within 4 to 6 wk. To maximize maize yield and provide satisfactory weed control, a range of 100 to 150 g a.i. ha^−1^ of isoxaflutole is recommended, depending on the soil characteristics, weather, and weed species present at the experimental site. Based on the results, isoxaflutole PRE has good potential for weed control in maize in China.

## Introduction

Maize was planted on more hectares than any other crops in China from 2010 to 2014, with an average of 35 million ha planted per year and yield averaging 5,779 kg per ha per year^1^. Weed competition can have a significant effect on crop yield. Potential grain yield loss in maize due to weeds was estimated to be 37 to 44%^2^. *Setaria viridis* (L.) Beauv., *Eleusine indica* (L.) Gaertn., *Digitaria sanguinalis* (L.) Scop., *Echinochloa crus-galli* (L.) Beauv., *Portulaca oleracea* L., *Amaranthus retroflexus* L., *Cyperus rotundus* L., *Cirsium arvense* (L.) Scop., and *Abutilon theophrasti* Medik. are major troublesome weeds in Chinese maize production systems^3^. These weeds can cause substantial yield reduction if not satisfactorily controlled.

In China, herbicides have been the main means of weed control for at least 35 years^4^, and today high-yielding agriculture remains heavily dependent on chemical weed control^5^. Currently, many herbicides have been used for weed control in maize, such as acetochlor, atrazine, nicosulfuron, thifensulfuron, and mesotrione^3^. Unfortunately, failure of the common herbicides has been occurring in many parts of China due to herbicide resistance evolution. For example, Zhou *et al*.^6^ reported that the sensitivity of *D. sanguinalis* to atrazine and acetochlor has been gradually declining in Henan province since 2001. In recent years, *E. crus-galli* and *A. retroflexus* have evolved resistance to nicosulfuron and thifensulfuron or atrazine, respectively, in Heilongjiang province^7^. Therefore, an alternative herbicide with high efficacy, broad-spectrum weed control and high level maize safety is needed to control these harmful weeds in maize fields.

Isoxaflutole is a selective PRE and early POST herbicide belonging to the isoxazole herbicide chemistry family^8^. Isoxaflutole is absorbed by plant roots or foliage, and it competitively inhibits the activity of 4-hydroxyphenylpyruvate dioxygenase (HPPD) blocking carotenoid biosynthesis in susceptible plants. Susceptible weed species treated with isoxaflutole initially show bleaching of the meristematic tissues followed by growth suppression and necrosis prior to plant death^9^. Isoxaflutole has been widely used in maize cultivation in Europe, North America, and Latin America for broadleaf and grass weed control. Previous research demonstrated that excellent control of both broadleaf and grass weeds was achieved when isoxaflutole was applied at 105 g a.i. ha^−1^ either alone or in combination with half of the normal use rate of other PRE herbicides such as acetochlor, alachlor, dimethenamid, or atrazine^10,11^.

Maize shows excellent tolerance to isoxaflutole. Bhowmik *et al*.^10^ and Vrabel *et al*.^12^ reported that maize was tolerant to PRE applications of isoxaflutole even at a rate of 158 g a.i. ha^−1^, with no adverse effects on grain yields. Nevertheless, several instances of isoxaflutole phytotoxicity in maize have been documented. These instances appear to be related to application timing^13^, high use rate^14^ and varied susceptibility of maize hybrids to isoxaflutole^15^. Environmental factors (wet and cold) and soil characteristics (organic matter content and soil type) can also lead to maize injury by isoxaflutole^16^.

Although its herbicide potential was identified in 1991^17^, isoxaflutole is still a new active ingredient in the Chinese market. Little research is available pertaining to the control of problematic weeds in maize with isoxaflutole applied PRE in China. Therefore, with the diversity of Chinese maize hybrids and associated weeds, the objectives of this study are (1) to determine the relative control efficacies of isoxaflutole against 12 weed species and the tolerance of 21 maize hybrids to isoxaflutole; (2) to investigate isoxaflutole selectivity between a common Chinese maize hybrid and two common weed species; and (3) to evaluate and identify isoxaflutole appropriate use rates under field conditions.

## Materials and Methods

### Weed species and maize hybrids

The weed species and maize hybrids used in the greenhouse study were presented in Tables 1 and 2. All weed seeds were collected in October 2013 from four uncultivated farms (farm GPS coordinates: Huangjiazhuang: 36.17°N, 117.16°E; Nanshanggao: 36.16°N, 117.17°E; Mazhuang: 35.97°N, 116.99°E; Baizi: 35.98°N, 117.13°E) in Tai’an city, China. These weed species are major troublesome weeds occurring in maize field, and the selected maize hybrids are on sale in Chinese agricultural market.

**Table 1:** Reductions in dry weight of weeds commonly found in maize fields in China 21 d after isoxaflutole treatment (DAT) relative to a nontreated control in a greenhouse study.

Trial weeds	Common name	Dry weight reduction (SE)^a^		F-statistic	P-value
		^____________^g ai ha^−1___________^			
		20	30		
		^___________^%^____________^			
*Setaria viridis* (L.) Beauv.	Green foxtail	83 (0.9)	87 (0.2)**	51.19	0.002
*Echinochloa crus-galli* (L.) Beauv.	Barnyardgrass	81 (0.6)	83 (0.8) NS	4.14	0.112
*Eleusine indica* (L.) Gaertn.	Goosegrass	89 (1.0)	96 (0.1)**	83.87	0.001
*Digitaria sanguinalis* (L.) Scop.	Large crabgrass	80 (1.6)	87 (0.5)**	59.08	0.002
*Cyperus rotundus* L.	Purple nutsedge	46 (3.3)	56 (2.2)*	14.57	0.019
*Cirsium arvense* (L.) Scop.	Canada thistle	59 (2.1)	63 (1.6) NS	3.33	0.142
*Amaranthus retroflexus* L.	Redroot pigweed	88 (1.6)	92 (0.8)*	9.12	0.039
*Abutilon theophrasti* Medik.	Velvetleaf	94 (0.4)	96 (0.5)*	11.21	0.029
*Portulaca oleracea* L.	Common purslane	97 (0.1)	98 (0.2)***	82.71	0.001
*Solanum nigrum* L.	Black nightshade	86 (0.9)	89 (1.6) NS	6.95	0.058
*Eclipta prostrata* (L.) L.	Eclipta	95 (0.1)	96 (0.5)*	12.91	0.023
*Xanthium strumarium* L.	Common cocklebur	94 (0.2)	96 (0.9)*	13.81	0.021

^a^Significant differences between the two PRE rates according to Fisher’s protected LSD test. *significant at P < 0.05; **significant at P < 0.01; ***significant at P < 0.001; NS, not significant.

**Table 2:** Plant height inhibition (%) and dry weight reduction (%) in maize 21 d after isoxaflutole treatment (DAT) relative to a nontreated control in a greenhouse study.

Corn hybrid	Plant height reduction (SE)^a^		F-statistic	P-value	Corn injury rating (SE)^a,b^		F-statistic	P-value
	^__________^g ai ha^−1____________^				^_______^g ai ha^−1________^			
	110	220			110	220		
	^_____________^%^______________^				^____________^%^___________^			
Denghai 3	13 (1.4)	17 (1.1)*	10.85	0.030	36 (1.9)	52 (2.9)**	42.20	0.003
Lubainuo 1	12 (0.8)	14 (0.9)*	9.81	0.035	33 (2.1)	44 (3.5)*	15.27	0.017
Zhengdan 958	8 (1.2)	13 (1.7)**	10.43	0.032	20 (1.7)	39 (4.2)**	35.03	0.004
Jinbei 288	8 (0.9)	8 (0.9) NS	0.92	0.389	18 (1.6)	27 (2.2)**	21.86	0.009
Denghai 6213	8 (0.7)	18 (0.4)***	292.86	0.000	19 (2.7)	55 (3.4)***	149.55	0.000
Zhenghuangnuo 2	8 (0.9)	13 (0.5)**	46.12	0.002	18 (5.3)	45 (3.8)***	173.25	0.000
Huanuo 1	7 (1.9)	13 (1.9)*	12.62	0.024	12 (2.2)	41 (3.6)**	95.34	0.001
Denghai 661	6 (1.8)	20 (0.9)**	103.82	0.001	10 (1.3)	63 (3.4)***	422.98	0.000
Denghai 3622	6 (0.4)	7 (0.6)*	10.14	0.033	10 (1.9)	24 (1.7)**	63.27	0.001
Jinlaiyu 5	5 (0.4)	13 (1.5)**	51.68	0.002	8 (1.8)	39 (2.9)***	167.15	0.000
Ziyu 2	5 (0.1)	7 (0.6)**	30.08	0.005	9 (0.8)	18 (3.0)*	15.10	0.018
Lunuo 6	5 (0.7)	6 (1.9) NS	1.63	0.271	7 (1.8)	13 (2.2)*	10.16	0.033
Shannong 8	5 (0.7)	7 (1.9) NS	1.91	0.240	8 (1.7)	15 (2.0)*	13.278	0.022
Denghai 605	5 (0.6)	13 (0.9)**	27.67	0.006	6 (2.5)	38 (3.2)***	127.41	0.000
Wuyue 21	5 (0.5)	9 (2.2)*	8.77	0.042	6 (1.5)	27 (2.3)***	113.85	0.000
Liaoyu 19	2 (0.7)	5 (0.2)**	30.63	0.005	4 (1.8)	7 (2.0) NS	3.31	0.143
Ludan 984	3 (1.2)	5 (1.6) NS	1.91	0.239	5 (2.3)	8 (1.1) NS	2.58	0.180
Jinlai 98	3 (0.9)	11 (0.5)***	179.32	0.000	5 (0.5)	26 (3.3)**	76.56	0.001
Lainong 14	3 (0.3)	5 (0.3)**	30.79	0.005	0	0 NS	ND^c^	ND
Jundan 29	3 (0.5)	3 (0.8) NS	1.44	0.297	0	0 NS	ND	ND
Jinwang 3	2 (0.3)	4 (0.9) NS	5.57	0.078	0	0 NS	ND	ND

^a^Significant differences between the two PRE rates according to Fisher’s protected LSD test. *significant at P < 0.05; **significant at P < 0.01; ***significant at P < 0.001; NS, not significant. ^b^Injury rating scale: 0 = consist with contrast treatment, 0~30% = cotyledon and minority of functional leaves showed whitening except new-born leaves, 30~60% = cotyledon, minority of functional leaves and new-born leaves showed whitening, 60~100% = majority of the plants showed serious whitening symptoms, some plants even showed necrosis, 100% = all plants showed whitening symptoms and necrosis. ^c^ND, not determined.

### Herbicide formulation

Isoxaflutole with 97.2% purity (provided by Qingdao Nongguan LLC, Shandong, China) was dissolved in a proper volume of acetone (<1%, v/v) and diluted with a 1% aqueous solution of Tween 80 (Solarbio Life Sciences, Beijing, China) to obtain the required application rates for the greenhouse study. Isoxaflutole with 97.2% purity was processed into a suspension concentrate formulation (24% SC) by Qingdao Nongguan LLC, and the isoxaflutole SC, mesotrione (Callisto®, 9% SC, Syngenta, Shanghai, China), and acetochlor (Acetochlor®, 50% EC, Rainbow Chemical, Shandong, China) were dissolved and diluted with water to obtain the required application rates for the field experiments.

### Greenhouse experiment design

All the greenhouse experiments were conducted from October 2014 to May 2015 in a controlled greenhouse at Shandong Agricultural University, Tai’an, China (36.20°N, 117.13°E). The greenhouse conditions were maintained as follows: 30 ± 2/21 ± 2 C (day/night), 75 ± 5% relative humidity, 14/10 h (light/dark) photoperiod achieved with natural light and augmented with supplemental lights (400-W high-pressure sodium lamp, 400 W, Philips, Amsterdam, the Netherlands), and 1,400 µmol s^−1^ m^−2^ average photosynthetic photon flux density (PPFD) across replications for daytime hours. Seeds from 12 weed species and 21 maize hybrids were germinated in a growth chamber (Model RXZ, Ningbojiangnan Instrument Factory, Zhejiang, China). Pre-germinated seeds were sown in 20-cm-diameter, 11-cm-deep plastic pots containing loam soils (pH 6.4 and 1.7% organic matter). Each pot was placed in a plastic tray and watered every other day. The PRE treatments of isoxaflutole were applied at 24 h after planting. Herbicides were applied using an auto-spraying tower (Model ASS-4, National Agricultural Information Engineering and Technology Centre of China, Beijing, China) with a Teejet-9503EVS flat-fan nozzle calibrated to deliver 450 L·ha^−1^ at 275 kPa. After treatment, all the pots were return to the controlled greenhouse for subsequent cultivation. The experimental design of all the greenhouse studies was a completely randomized design with three replications per treatment, and the experiments were conducted twice.

#### Effectiveness of weed control

Twenty five and 20 seeds were planted per pot for the grass and broadleaf weeds, respectively. The PRE treatment of isoxaflutole were applied as an active ingredient (a.i.) at the rate of 20 and 30 g a.i. ha^−1^, and a nontreated control was established for each weed species. Other experimental conditions were the same as described in the greenhouse experiment design. At 21 d after treatment (DAT), surviving weed plants were counted, cut at the soil surface, oven-dried at 80 °C for at least 72 h, and the dry weight recorded^18^.

#### Maize hybrid tolerance

Five seeds of maize were sown per pot. The PRE treatments of isoxaflutole at 110 and 220 g a.i. ha^−1^ were applied, and a nontreated control used for each maize hybrid. Other experimental conditions were the same as previously stated in the greenhouse experiment design. At 21 DAT, plant height was measured and recorded. Moreover, visual estimates of herbicide damage to maize seedlings were also recorded by an independent assessor using a scale of 0 to 100% (0 = no damage, 100 = total death)^19^.

#### Selectivity index (SI)

The ratio between the rates that caused 10% of growth reduction to the crop and 90% of growth reduction to the weed was used as a selectivity index^20^. Zhengdan 958 is one of the most popular maize hybrids in the maize growing region in China^21^, and *E. indica* and *P. oleracea* are the most common and troublesome weed species in Chinese maize production systems^3^. To quantify the selectivity between the tolerant maize and the weed species, Zhengdan 958 was treated with PRE applications at 0, 60, 180, 240, 300, and 360 g a.i. ha^−1^, and *E. indica* and *P. oleracea* were simultaneously treated with PRE applications at 0, 1.25, 2.5, 5, 10, and 20 g a.i. ha^−1^. Other experimental conditions were the same as previously stated. After 21 d of cultivation, the plant dry weights were obtained. The rates of isoxaflutole required for 10% and 50% reductions in the shoot dry weight of the maize (IC_10_ and IC_50_ values) and 50% and 90% reductions in the shoot dry weight of the weeds (IC_50_ and IC_90_ values) were calculated from non-linear regression equations (Table 3).

**Table 3:** Rates of isoxaflutole application causing 10% and 50% reduction in the growth of Zhengdan 958, and 50% and 90% reductions in the growth of *Eleusine indica* and *Portulaca oleracea* as well as the selectivity index (SI) between Zhengdan 958 and the two weeds 21 d after treatment (DAT) in a greenhouse study.

Trial plants	IC value (SE)^a^			SI^b^
	IC_10_	IC_50_	IC_90_	
	^_______________^g a.i. ha^−1________________^			
Zhengdan 958	232.4 (10.1)	292.4 (14.3)	ND^c^	ND
*Eleusine indica*	ND	2.2 (0.1)	19.3 (1.0)	12.0
*Portulaca oleracea*	ND	2.0 (0.1)	18.8 (0.8)	12.4

^a^IC, inhibitory concentration. ^b^SI, selectivity index. Selectivity index was calculated according to equation 2: *SI* = *IC*_*10(maize)*_/*IC*_*90(weed)*_. ^c^ND, not determined.

### Field experiment design

Field experiments were conducted twice during the maize growing seasons in 2015 and 2016 at the Research Farm of Shandong Agricultural University (36.17°N, 117.16°E, altitude 139 m, with a yearly average precipitation of 697 mm), Tai’an, China. The soil type at the test site was a loam (brown earth, a type of Luvisol) with 1.7% organic matter and a pH of 7.1, and the area of brown earth was 25 million hectares in China^22^. The area was heavily infested with *S. viridis*, *D. sanguinalis*, *A. retroflexus*, and *A. theophrasti*. The monthly air temperature and precipitation at the experimental site in 2015 and 2016 are presented in Table 4. In both years, the experimental site was ploughed with mouldboard, disked and finally smoothed with a land leveller prior to maize planting. Zhengdan 958 maize was mechanically planted at a depth of 3 cm and a density of 75,000 seeds ha^−1^ on June 26, 2015 and June 20, 2016. In accordance with the local maize production practices in Tai’an, necessary fertilizers were applied at planting or topdressed.

**Table 4:** Monthly air temperature and total precipitation at the experimental site at Tai’an, Shandong, China during the maize growing season in 2015 and 2016.

Month	Air temperature (°C)						Total precipitation (mm)	
	Maximum		Minimum		Mean			
	2015	2016	2015	2016	2015	2016	2015	2016
June	35.9	34.3	15.6	15.8	25.0	25.1	79.9	167.8
July	38.2	36.0	18.2	17.6	27.2	27.2	86.0	219.7
August	33.9	34.5	16.6	12.7	25.3	26.2	138.2	228.9
September	29.9	32.8	8.4	9.3	21.5	22.0	13.3	16.7

All treatments were established in a randomized complete block design with four replications. Plots consisted of 8 rows, 5 m in length with 0.50 m row spacing. The treatments consisted of four rates at 100, 125, 150, and 250 g a.i. ha^−1^ of isoxaflutole; a single rate of mesotrione; a tank mix of isoxaflutole plus acetochlor; a hand-weeded control (using a hand hoe at 15, 30, and 45 d after planting, DAP); and a weedy control, providing a total of 8 treatments, as shown in Table 5. The PRE applications were made on June 27, 2015 and June 21, 2016. All herbicides were applied in 450 L ha^−1^ of water with a backpack sprayer (Bellspray Inc., Opelousa, LA) equipped with a single 8002 VS nozzle (Teejet Technologies, Wheaton, IL).

**Table 5:** Visual estimates of percent weed control following different herbicide treatments in 2015 and 2016 at Tai’an, Shandong, China.

Treatment	Timing	Dose	Percent weed control^a^							
			*S. viridis*		*D. sanguinalis*		*A. retroflexus*		*A. theophrasti*	
			2015	2016	2015	2016	2015	2016	2015	2016
		g a.i. ha^−1^	^______________________^%^________________________^							
Isoxaflutole	PRE	100	88^c^	91^d^	87^d^	89^d^	92^b^	95^b^	86^a^	89^a^
Isoxaflutole	PRE	125	93^b^	95^c^	92^c^	93^c^	97^ab^	99^ab^	88^c^	92^c^
Isoxaflutole	PRE	150	96^b^	98^b^	96^b^	96^b^	100^a^	100^a^	92^b^	93^b^
Isoxaflutole	PRE	250	99^a^	99^a^	98^a^	99^a^	100^a^	100^a^	94^a^	95^a^
Mesotrione	PRE	225	59^d^	64^e^	88^c^	93^d^	96^ab^	98^ab^	91^a^	94^a^
Isoxaflutole + acetochlor	PRE	100 + 900	96^b^	97^bc^	96^b^	98^b^	100^a^	100^a^	94^a^	96^a^
Hand weeding	—	—	—	—	—	—	—	—	—	—
Weedy control	—	—	—	—	—	—	—	—	—	—

^a^Visual estimates of percent weed control were recorded 30 d after treatment (DAT) using a scale of 0 to 100% where 0 = no weed control and 100 = complete weed control. Means followed by the same letter are not significantly different (P ≤ 0.05).

Visual estimates of percent weed control were recorded 30 DAT using a scale of 0 to 100%, where 0 = no weed control and 100 = complete weed control. The visual crop injury was evaluated at 5, 15, and 30 DAT using a scale of 0 to 100%, where 0 = no crop injury and 100 = plant death^23^. Corn yields were determined by harvesting the centre two rows of each plot with a plot combine harvester. The seed weight was adjusted to 13% moisture.

### Data Analysis

The data sets from the repeated experiments in the greenhouse were analysed by ANOVA with the general linear model procedure using SPSS software (v.17.0; IBM Corporation, Armonk, NY). The statistical analysis indicated that there was no significant interactions (P > 0.05) between treatment and year, and thus the data were pooled across years for subsequent analyses. All regression analyses were conducted using SigmaPlot software (v.13.0; Systat Software Inc., CA, USA). The rate-response curves were obtained by non-linear regression analysis using the logistic response equation (equation 1) proposed by Seefeldt *et al*.^24^. The fitted model was as follows:

1 $$Y=C+\frac{(D-C)}{[1+(\frac{{\rm{x}}}{I{C}_{50}}{)}^{b}]}$$

where *C* is the lower limit of the response, *D* is the upper limit of the response, *x* is the herbicide application rate, *IC*_50_ is the rate causing 50% of the maximum response and *b* is the slope of the curve at the *IC*_50_.

Based on the regression parameters, the *IC*_10_, *IC*_50_, and *IC*_90_ herbicide selectivity values were calculated. The *SIs* of isoxaflutole were calculated with equation 2 as follows:

2 $$SI=\frac{I{C}_{10}(maize)}{I{C}_{90}(weed)}$$

where *IC*_10_ equals a 10% effect on Zhengdan 958 and *IC*_90_ equals a 90% effect on the trial weeds.

The data from the field experiments were subjected to ANOVA, and means were separated using Fisher’s protected LSD test at the P < 0.05 significance level. When ANOVA revealed no significant (P > 0.05) difference in two years’ treatment interaction, data were pooled over years.

## Results and Discussion

### Greenhouse Experiments

#### Effectiveness of Weed Control

Isoxaflutole showed a high efficacy for common broadleaf and grass weed control in the greenhouse study. At 20 g a.i. ha^−1^, PRE application of isoxaflutole resulted in high efficacy^3,25^ (>85% reduction in dry weight) against 7 of the weeds that were tested in this experiment, including *E. indica*, *A. retroflexus*, *A. theophrasti* and *P. oleracea*, *S. nigrum*, *E. prostrata*, and *X. strumarium* (Table 1). When treated at 30 g a.i. ha^−1^, the number of weed species that was controlled increased to 9 (Table 1). Isoxaflutole at both rates displayed good efficacy (>90%) on some large-seeded broadleaf weeds, such as *X. strumarium* and *A. theophrasti*, which was consistent with previous findings^26,27^. PRE herbicides such as S-metolachlor and alachlor provide high efficacy against a large number of small-seeded weeds but offer only limited control of large-seeded broadleaf weeds^28^. The capability of controlling some large-seeded broadleaf weeds may make isoxaflutole a better choice than other common PRE herbicides for weed control in corn in China.

The weed species exhibited variable sensitivity to isoxaflutole. *P. oleracea* was the most susceptible, followed by *E. prostrata*, *A. theophrasti*, *X. strumarium*, *E. indica*, *A. retroflexus*, *S. nigrum*, *S. viridis*, *E. crus-galli*, *D. sanguinalis*, *C. arvense*, and *C. rotundus*. Similarly, Bhowmik *et al*.^26^ reported that weed species differed in their sensitivity to isoxaflutole and that *A. theophrasti* was the most susceptible. *C. rotundus* and *C. arvense* were more tolerant than other weeds in our experiment, and isoxaflutole could not control them effectively (<64%) through PRE application (Table 1). Fortunately, acetochlor and triazine herbicides are still effective in controlling these two abovementioned weeds. To provide extended control of the tolerant weed species, combining isoxaflutole with acetochlor or atrazine could be a potential practice.

In some regions of China, *D. sanguinalis* and *A. retroflexus* have evolved resistance to atrazine in maize fields^29^. Luckily, a PRE application of 30 g a.i. ha^−1^ of isoxaflutole in the greenhouse was highly effective (>85%) in controlling these two weed species (Table 1). Similarly, Bhowmik *et al*.^26^ reported that isoxaflutole provided complete control of *D. sanguinalis* at 36 g a.i. ha^−1^ in a greenhouse study. The application of isoxaflutole in agricultural production could help control atrazine-resistant weeds.

#### Maize Hybrid Tolerance

Isoxaflutole was found to be safe^23^ for most of the 21 tested maize hybrids under PRE applications in the greenhouse experiment, but there were some variations between maize hybrids. When treated PRE at 110 g a.i. ha^−1^, Denghai 3 and Lubainuo 1 were sensitive to isoxaflutole, and plant height reductions were 13% and 12%, respectively. The herbicide damage to these two hybrids was beyond 30% (Table 2). Isoxaflutole was safe on the other 19 maize hybrids, whose reductions in plant height were at or below 8% and whose herbicide damage was at or below 20%. Bhowmik *et al*.^26^ reported that maize was tolerant to PRE application of isoxaflutole in the greenhouse, and Sprague *et al*.^15^ stated that the susceptibility of different maize hybrids to isoxaflutole is varied, which was in accordance with our research.

However, when treated PRE at 220 g a.i. ha^−1^, almost half of the 21 maize hybrids, such as Denghai 3, Lubainuo 1, and Denghai 661, were sensitive to isoxaflutole, and plant heights inhibited by more than 10% (Table 2). Furthermore, the herbicide damage of these maize hybrids was at least 26% (Table 2). This result indicated that the isoxaflutole rate used in maize fields should not exceed the recommended rates (79 to 131 g a.i. ha^−1^ for medium- and fine-textured soils^30^), as this herbicide will damage common maize hybrids in China. Nevertheless, the present research only focused on the susceptibility of different maize hybrids to isoxaflutole based on a specific soil type. China is a very large country with many different soil types and environmental conditions. Maize can be planted in various soils with different environmental conditions. Importantly, isoxaflutole can cause significant injury when maize is grown on sandy soils and experiences cold, wet soil conditions^16,27^. Further experiments should be carried out in different regions around China to determine the effects of soil properties and environmental factors on isoxaflutole injury to specific maize hybrids.

#### Selectivity Index (SI)

**Figure 1:**
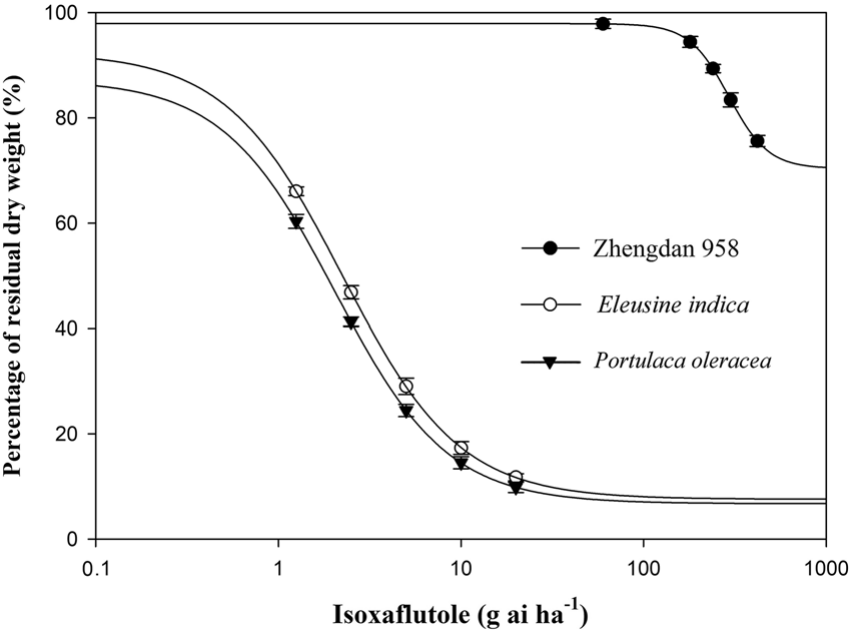
Percentages of residual dry weight of different trial plants at 21 d after treatment (DAT) were fitted to a functional, four-parameter logistic curve model (Equation 1) with increasing rates of isoxaflutole application obtained from the greenhouse study. The fitted equations were as follows: Zhengdan 958, Y = 70.4 + 27.5/[1 + (x/292.4)^−4.0^], R^2^ = 0.99; *Eleusine indica*, Y = 7.5 + 84.9/[1 + (x/2.2)^−1.4^], R^2^ = 0.99; and *Portulaca oleracea*, Y = 6.7 + 80.6/[1 + (x/2.0)^−1.4^], R^2^ = 0.99.

Based on the glasshouse experiment results on the maize tolerance, a dose-response study was conducted to assess selectivity of Zhengdan 958 maize to PRE application of isoxaflutole. The high IC_50_ value clearly showed that PRE application was safe on Zhengdan 958 and that *E. indica* and *P. oleracea* were effectively controlled (Table 3). Additionally, the *P. oleracea* was more sensitive to isoxaflutole under PRE applications than *E. indica* (Fig. 1).

The selectivity index values for Zhengdan 958 maize, *E. indica*, and *P. oleracea* were also identified (Table 3). According to the report of Tind *et al*.^31^, the more the SI increases past 1.0, the more selective the compound between the crop and weeds. Bartley^32^ stated that a compound could safely be used on a crop when the SI was greater than 2.0. In the present study, isoxaflutole was found to be safe for maize against *E. indica* and *P. oleracea* when applied PRE with SI values of 12.0 and 12.4, respectively. The results indicated that isoxaflutole could safely be used in Zhengdan 958 maize on the soil type used in the greenhouse study.

### Field Experiment

#### Field weed control efficacy

The experimental site was composed of *S. viridis*, *D. sanguinalis*, *A. retroflexus*, and *A. theophrasti* weed species in both experimental years. The weed density was estimated based on the weedy control plots. The average density in 2015 and 2016 for *S. viridis* was 30 and 32 plants m^−2^, 21 and 23 plants m^−2^ for *D. sanguinalis*, 15 and 14 plants m^−2^ for *A. retroflexus*, and 6 and 8 plants m^−2^ for *A. theophrasti*, respectively. Rainfall during the 14-d period after PRE herbicide treatment differed between the two years. Rainfall accumulations were greater than 97.4 mm during the first 7 d after PRE herbicide treatment in 2016, but there was only 8.9 mm of rainfall that occurred during the 7 DAT in 2015 (data not shown).

The visual estimates of percent weed control for treatments are presented in Table 5, and the data were similar to the results of the greenhouse study. Overall, the weed densities of the four weed species observed decreased as the rates of isoxaflutole increased from 100 to 250 g a.i. ha^−1^ in both years (Table 5). All weed species infesting the maize field were controlled by more than 85% by isoxaflutole PRE at all test rates. The tank mixture of isoxaflutole at 100 g a.i. ha^−1^ with acetochlor at 900 g a.i. ha^−1^ increased the control of all four weeds to greater than 93%. Young *et al*.^27^ reported that isoxaflutole must be combined with other herbicides to achieve consistent control of a broad spectrum of weed species. The combined results of the field and greenhouse studies indicate isoxaflutole has a good efficacy on weed control. Moreover, a tank mix of isoxaflutole with acetochlor may have great potential to provide extended control of many difficult-to-control weeds, such as *C. rotundus* and *C. arvense*.

In this experiment, the efficacy of all herbicide treatments was higher in 2016 than in 2015. The differences in control between years may result from more rainfall 1 to 7 DAT in 2016 (97.4 mm) than in 2015 (8.9 mm). Moyer^33^ reported that the soil moisture content influenced herbicide concentration in the soil solution and efficacy of soil-applied herbicides. Under increasing rainfall and soil moisture conditions, weed control with isoxaflutole was improved according to our data.

#### Field maize tolerance

Where noted, the maize injury displayed as bleaching and twisting of newly developed tissue followed by stunting, which are common symptoms associated with isoxaflutole^34^. Maize injury data in 2015 and 2016 were presented in Table 6. Based on the visual ratings, temporary maize injuries occurred with isoxaflutole PRE at 125, 150, and 250 g a.i. ha^−1^ in both years. Injury did not exceed 10% with PRE applications at 125 and 150 g a.i. ha^−1^ in both years, but reached 16% and 21% with PRE application at 250 g a.i. ha^−1^ in 2015 and 2016, respectively. Injury in 2016 was more severe than that in 2015, when 97.4 mm of rainfall accumulated within 7 DAT. Bleaching of the leaves occurred in both years, but this did not reduce grain yields due to plant recovery.

**Table 6:** Visual estimates of injury to maize and maize yields following different herbicide treatments at Tai’an, Shandong, China in 2015 and 2016.

Treatments	Timing	Rate	Crop injury^a,b^						Maize yield^b,c^		Yield growth rate^c^	
			5 DAT		15 DAT		30 DAT					
			2015	2016	2015	2016	2015	2016	2015	2016	2015	2016
		g a.i. ha^−1^	^______________________^%^________________________^						^________^kg ha^−1________^		^_________^%^_______^	
Isoxaflutole	PRE	100	0	0 NS	0	0 NS	0	0 NS	8834^d^	9137^d^*	12.1^d^	13.7^d^
Isoxaflutole	PRE	125	2	5*	1	3*	0	0 NS	9127^c^	9397^c^*	15.8^c^	16.9^c^
Isoxaflutole	PRE	150	7	10*	5	9*	0	0 NS	9313^b^	9627^b^*	18.1^b^	19.8^b^
Isoxaflutole	PRE	250	16	21*	12	17*	1	2*	9262^bc^	9425^c^*	16.8^c^	17.3^c^
Mesotrione	PRE	225	0	0 NS	0	0 NS	0	0 NS	8866^d^	9167^d^*	12.5^d^	14.1^d^
Isoxaflutole + acetochlor	PRE	100 + 900	0	0 NS	0	0 NS	0	0 NS	9297^b^	9590^b^*	17.9^b^	19.3^b^
Hand weeding	—	—	—	—	—	—	—	—	9818^a^	10055^a^*	24.6^a^	25.1^a^
Weedy control	—	—	—	—	—	—	—	—	7883^e^	8036^e^*	—	—

^a^Visual crop injury was evaluated at 5, 15, and 30 d after treatment (DAT) on a 0 to 100% scale, with 0% representing no injury and 100% representing plant death. ^b^Significant differences between the maize injuries or maize yields of both years according to Fisher’s protected LSD test at P < 0.05. *significant; NS, not significant. ^c^Means followed by the same letter are not significantly different (P ≤ 0.05).

Instances of isoxaflutole phytotoxicity in maize attributed to several factors, which have been mentioned above. Bhowmik *et al*.^26^ observed maize injury in fine-textured soil (Hadley fine sandy loam) when isoxaflutole was applied at 210 g a.i. ha^−1^, and Sprague *et al*.^15^ also reported maize injury in coarse-textured soils (low clay and organic matter) from rates of 158 g a.i. ha^−1^ of isoxaflutole in Michigan. The temporary injury to Zhengdan 958 maize observed in the current study may be related to the high use rate, medium-textured soil (43% sand) and rainfall shortly after herbicide application, which may improve the activation and uptake of isoxaflutole. Reducing isoxaflutole rates, or applying the herbicide several weeks before planting could potentially reduce phytotoxicity to maize.

#### Field grain yield

The maize grain yield increased with increasing the isoxaflutole rate from 100 to 150 g a.i. ha^−1^ at Tai’an city in both years, as compared to the control without herbicide application (Table 6). For example, increasing the isoxaflutole rate from 0 to 150 g a.i. ha^−1^ resulted in an 18.1% yield increase in 2015. Maize yields varied with year and were likely affected by weed control and rainfall. In 2015 and 2016, rainfall during June and July was 165.9 and 387.5 mm, respectively (Table 4), and maize yields in 2016 were higher than in 2015 (Table 6). However, the yield from 250-g a.i. ha^−1^ treatment was lower than from 150 g a.i. ha^−1^ in both years. Optimum maize yields were achieved with isoxaflutole rates ranging from 100 to 150 g a.i. ha^−1^ across years (Table 6), and maize treated with isoxaflutole at 100 g a.i. ha^−1^ plus acetochlor at 900 g a.i. ha^−1^ produced yields similar to that of maize treated with the isoxaflutole at 150 g a.i. ha^−1^. Therefore, to maximize maize yields and provide satisfactory weed control, a range of 100 to 150 g a.i. ha^−1^ of isoxaflutole is recommended according to our study.

Uncontrolled weeds reduced yield by more than 24% in the weedy check plots compared to the hand-weeded plots in both years, indicating that ineffective weed control will result in greater reduction in maize yield. None of the herbicide treatments led to superior grain yields compared to hand weeding. The results revealed that although hand weeding during the growth season was an effective treatment (highest yield among eight treatments), the cost and labour requirement may make it economically unjustifiable^35,36^. Although not tested in the present study, a combination of herbicide and hand weeding may result in efficient and economical weed control in maize.

## Conclusion

In summary, the results obtained from both the greenhouse and field experiments demonstrate that isoxaflutole has great potential as a selective PRE herbicide for weed control in Chinese maize production. Especially in situations where weeds have evolved resistance to triazine and amide herbicides, and this PRE herbicide will be a powerful tool for farmers to effectively manage herbicide resistance in China. In addition, although this study showed that most weed species were still sensitive to common herbicides, more attention should be paid to avoid the occurrence and evolution of resistance. Priority should be given to delay the evolution of herbicide resistance in *D. sanguinalis* and *A. retroflexus*. Reducing the herbicide selection pressure is essential for delaying the resistance evolution by applying herbicides with different application timings or different modes of action in mixture or in rotation.

### Data availability

The datasets generated during and/or analysed during the current study are available from the corresponding author on reasonable request.
