# Publisher Correction: Greenhouse and field evaluation of isoxaflutole for weed control in maize in China

**DOI:** 10.1038/s41598-017-16209-4

**Published:** 2017-11-23

**Authors:** Ning Zhao, Lan Zuo, Wei Li, Wenlei Guo, Weitang Liu, Jinxin Wang

**Affiliations:** Key Laboratory of Pesticide Toxicology and Application Technique, College of Plant Protection, Shandong Agricultural University, Tai’an, 271018 China

*Scientific Reports* **7**:12690; doi:10.1038/s41598-017-12696-7; Article published online 04 October 2017

The original HTML version of this Article contained an error in the Materials and Methods section under subheading ‘Data Analysis’.

“The fitted model was as comment = “The superscript &quot;b&quot; was missed in our manuscript. We have added it to the equation 1.“follows:”

now reads:

“The fitted model was as follows:”

This error has now been corrected in the HTML version of the Article; the PDF version was correct from the time of publication.

